# The Usefulness of MRI Dynamic Flow Sequences in Differentiating High- and Low-Flow Vascular Malformations

**DOI:** 10.3390/jcm12010101

**Published:** 2022-12-23

**Authors:** Natalia Krystyna Majewska, Marcin Stański, Joanna Ryczkowska, Jacek Wątorek, Alicja Stańska, Mateusz Wykrętowicz, Małgorzata Pyda, Katarzyna Katulska

**Affiliations:** 1 Department of General Radiology and Neuroradiology, Poznan University of Medical Sciences, 60-355 Poznań, Poland; 2 Department of Endocrinology, Metabolism and Internal Diseases, Poznan University of Medical Sciences, 60-355 Poznań, Poland; 3 1st Department of Cardiology, Poznań University of Medical Sciences, 61-848 Poznań, Poland

**Keywords:** vascular malformations, arteriovenous malformations, magnetic resonance imaging

## Abstract

Peripheral arteriovenous malformations (PVMs) can be classified into high-flow malformations (HFVMs) and low-flow malformations (LFVMs). Adequate distinguishment is crucial for therapeutic decision and can be done using dynamic contrast-enhanced MRI (DCE-MRI). The main aim of this retrospective study was to determine the diagnostic value of quantitative DCE-MRI ratios for differentiation between HFVM and LFVM, their optimal cut-off points, and predictive values. DCE-MRI time-resolved angiography with stochastic trajectory (TWIST) examinations of 90 patients with PVMs were included [28 HFVM (31%), 62 LFVM (69%)]. The measurements of artery-lesion time, maximum lesion enhancement, slope of the enhancement curve, and maximum percentage increase of signal intensity (SI) were obtained. The optimal cut-offs for HFVMs calculated using the Youden index were: for slope of enhancement curve < 8.7 s (sensitivity of 86%, specificity of 89%), artery-lesion time ≤ 5.6 s (sensitivity of 89%, specificity of 77%), time to maximum enhancement ≤ 30 s (sensitivity of 94%, specificity of 100%), and maximum percentage enhancement of the lesion > 662% (sensitivity of 68%, specificity of 69%). To summarize, DCE-MRI is very valuable for differentiation between HFVM and LFVM, especially if quantitative measurements are done.

## 1. Introduction

Peripheral vascular malformations (PVMs) are congenital lesions that occur in 0.8–1% of the general population [1]. They have a wide spectrum of clinical manifestations, depending on size and localization. PVMs can be a cause of a severe aesthetic and functional impairment and, in the most extreme cases, may even lead to limb deformation or life-threatening heart failure [2]. Before choosing a proper form of treatment, it is always crucial to correctly classify the lesion according to its hemodynamic characteristics. The vascular malformations are categorized into high-flow and low-flow malformations (HFVMs and LFVMs), depending on the blood flow and the extent of arterio-venous shunting. HFVMs are typically managed with embolization, and LFVMs with percutaneous sclerotherapy [3,4].

An ultrasound examination, with a color and spectral doppler, may be helpful in the classification of subcutaneously located PVMs of relatively small size, with respect to the type of transducer. The flow characteristics and velocities may be evaluated. HFVMs are usually not susceptible to pressure and the nidus and feeding artery may be found; the flow characteristics of draining veins are arterialized. However, an ultrasound examination has strong limitations—it cannot accurately assess giant lesions and malformations located adjacent to bones or deep in the body, it is operator-dependent, and the operator variability is high [5].

Magnetic resonance imaging (MRI) has been widely used in PVMs classification. Currently, dynamic contrast-enhanced MRI (DCE-MRI) is considered the crucial sequence for this purpose [2,5]. It has high temporal and spatial resolution and enables plotting of the enhancement curve and quantitative assessments. However, until now, only a few studies focused on finding the proper DCE-MRI-derived ratios to distinguish between HFVMs and LFVMs. Moreover, the ratios examined by them were different. The papers determined the time interval to the maximum enhancement of the lesion or the time interval between the beginning of enhancement in the adjacent artery and in the PVM [6,7]. The measurements also included the maximum percentage enhancement of the lesion [8] and the slope of the enhancement curve [6]. However, all of these studies had important limitations, such as small sample size [6,7], the evaluation being made by two different radiologists, and a lack of a clearly defined reference method [8].

In this study, we sought to provide reliable data on all of the aforementioned ratios, with a relatively large sample size and an assessment by one experienced radiologist.

Moreover, it is interesting to consider the role of flow voids in this topic, as their presence can be used for differentiation between HFVMs and LFVMs and there is no need for an injection of a contrasting agent to correctly assess it. Flow voids are the signal loss in sites of rapid blood flow in spin echo (SE) sequences. They have been typically unseen in LFVMs, and their presence has been widely associated with arteriovenous HFVMs [9]. Although its specificity in the diagnosis of HFVM was characterized as high (90%), their sensitivity was found to be rather low (40%) [10,11], except for one recent study by Höhn et al., where the results were different (specificity of 96%, sensitivity of 100%) [9].

To sum up, our primary aim was to assess and compare the diagnostic value of different DCE-MRI-derived ratios in the differentiation between HFVM and LFVM [time-resolved magnetic resonance angiography with interleaved stochastic trajectories (TWIST) was used]. A secondary goal was to evaluate the value of flow voids, which can be seen on SE non-contrast scans, in the diagnosis.

## 2. Materials and Methods

This was a single-institution, retrospective study carried out in a tertiary university medical hospital. It was approved by the institutional Ethics Committee (ethics approval numbers 573/16 and 585/18). Informed consent was waived for all patients due to the retrospective nature of the study.

### 2.1. Patients

Ninety-seven patients who had MRI examinations, including a TWIST sequence, between 2010 and 2016 were enrolled in the study. The inclusion criterion was the presence of peripheral vascular malformation, confirmed by doppler ultrasound and/or digital subtraction angiography (DSA) and/or venography. There were two exclusion criteria: previous surgical treatment of vascular malformations (VM) and extensive respiration-induced artifacts on MRI scans which made further analysis impossible. Accordingly, 7 patients were excluded: 4 of them due to previous surgery and 3 due to artifacts. Eventually, a group of 90 patients was enrolled, 36 men (40%) and 54 women (60%).

**Figure 1:**
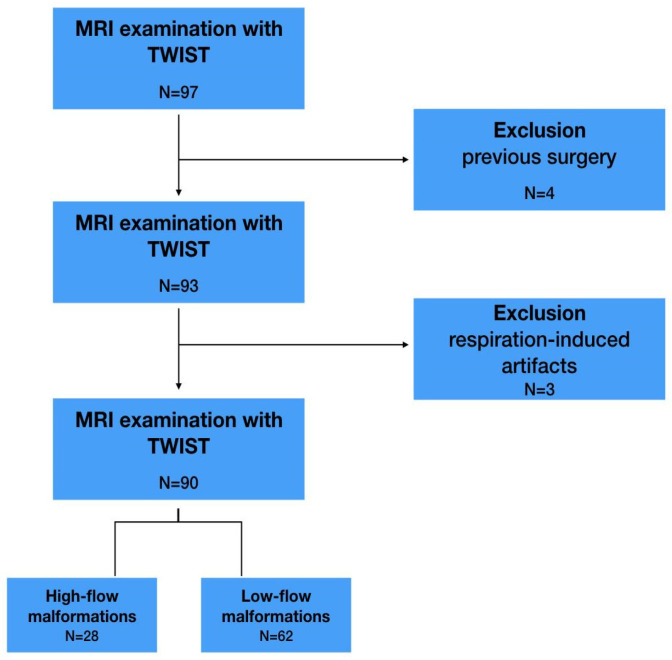
Flow chart of study identification, inclusion and exclusion criteria. MRI—magnetic resonance imaging, TWIST—time-resolved magnetic resonance angiography with interleaved stochastic trajectories.

The type of malformation (HFVM or LFVM) was defined based on imaging data used for inclusion in the study. This data were used as a reference in further analysis. Thus, 28 patients with HFVMs (31%) and 62 patients with LFVMs (69%) were included, as presented in Figure 1.

### 2.2. Image Acquisition

MR examination was performed using 1.5 T MAGNETOM Aera (Siemens Healthineers, Erlangen, Germany) or 1.5 T MAGNETOM Avanto (Siemens Healthineers, Erlangen, Germany). The coils were chosen according to the VM location: 4-channel Flex Small coil for hands or feet, and 8-channel Flex Large coil for forearms, arms, calves, and thighs. Chest lesions were examined using both Body Matrix and Spine Matrix coils. In midface malformation, a 12-channel Head/Neck coil was used.

Contrast-enhanced MRI protocol contained non-contrast T1-weighted (T1w) SE sequence (11 ms echo time (TE), 512 ms repetition time (TR), 358 × 448 field of view (FoV), 3 mm section thickness), non-contrast fat-saturated T2-weighted (T2w) SE sequence (2500–4000 ms TE, 45–55 ms TR, 220 × 256 FoV, 4 mm section thickness), and one or both of the following post-contrast sequences: T1w 3D gradient-echo (GE; 1.21 ms TE, 3.32 ms TR, 230 × 512 FoV, 1.5 mm section thickness) or T1w 3D GE fat-saturated VIBE (2.3 ms TE, 4 ms TR, 171 × 384 FoV, 3 mm section thickness). T1w 3D GE scans were obtained in 58 patients (64%), whereas T1w 3D GE fat-saturated VIBE images were taken in 83 patients (92%).

A TWIST sequence (0.9 ms TE, 2.3 ms TR, 246 × 352 FoV, 2.5 mm section thickness) was performed in the coronal plane before post-contrast T1w sequences. A single injection of 1.0 M gadobutrol (Gadovist, Bayer Healthcare, Berlin, Germany) at a dose of 0.1 mmol/kg and at an injection rate of 3.5 mL/s was performed, followed by an immediate 20 mL saline flush at the same rate. The injection was given using an electronic power injector (MEDRAD Spectris Solaris, Bayer Medical Care Inc., Indianola, IA, USA). Temporal resolution was between 3.5 s and 4.0 s per acquisition, depending on the size and location of the VM.

### 2.3. Image Evaluation

All readings were performed on a picture archiving and communication system (PACS)-integrated workstation (Syngo.via VB50, Siemens Healthineers, Erlangen, Germany) by a senior radiologist with 10 years of experience, blinded to clinical data and previous imaging. First, the presence of flow voids on T1w SE non-contrast images was reported.

**Figure 2:**
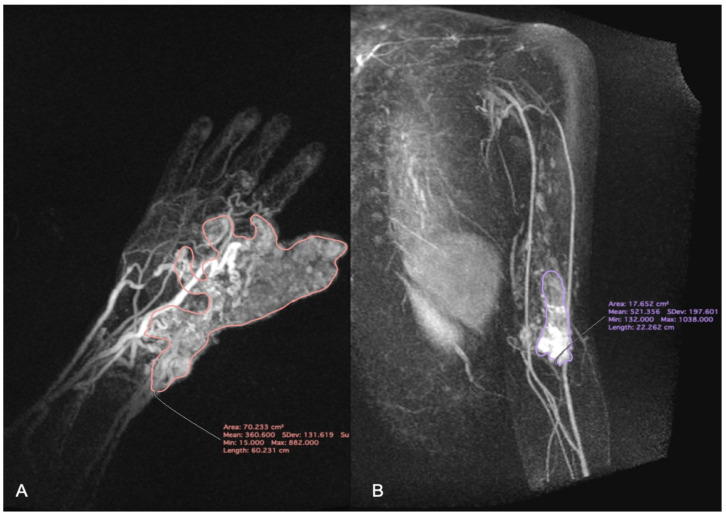
Time-resolved magnetic resonance angiography with interleaved stochastic trajectory (TWIST) maximum intensity projection (MIP) images. Vascular malformations (VMs) with the corresponding regions of interest (ROIs). (**A**) VM of the hand and wrist; (**B**) VM of the arm.

**Figure 3:**
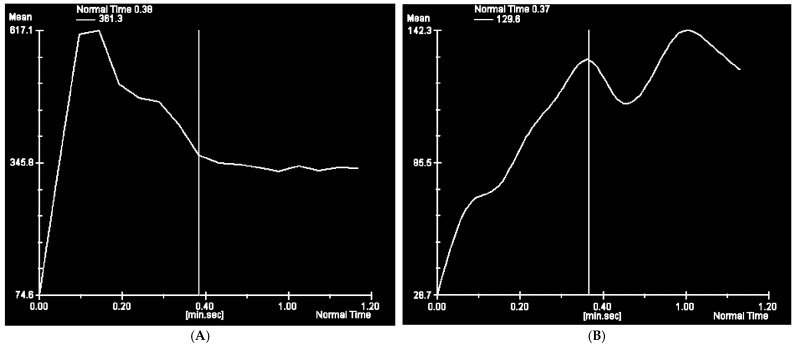
Time intensity curves for: (**A**) high-flow vascular malformations (HFVMs); (**B**) low-flow vascular malformations (LFVMs).

Then, the hemodynamic characteristic of VM was analyzed using TWIST maximum intensity projection (MIP) images. MIP images are obtained by projecting the voxel with the highest attenuation value on every view throughout the 3D image onto the 2D image. This method allows to display contrast-filled vessels preferentially and to get reconstructions similar to images obtained using the DSA [12]. The appropriate software (MeanCurve, Siemens Healthcare, Erlangen, Germany) was delivered by the workstation producer. First, the scan with the largest size of VM was chosen. The region of interest (ROI) was carefully, manually placed by the radiologist to contain the whole perimeter of the VM, as presented on Figure 2. Then it was duplicated on the rest of the scans. The signal intensity (SI) values inside all of the ROIs were measured and plotted versus the time of delay after contrast injection. The corresponding time intensity curve was calculated, as shown in Figure 3.

The maximum percentage increase of SI above the baseline was calculated using the following formula:

$$\mathrm{Maximum}\;\mathrm{percentage}\;\mathrm{increase}\;\mathrm{of}\;\mathrm{signal}\;\mathrm{intensity}=\frac{\mathrm{Maximum}\;\mathrm{signal}\;\mathrm{intensity}\;\mathrm{after}\;\mathrm{enhancement}\;-\;\mathrm{Signal}\;\mathrm{intensity}\;\mathrm{before}\;\mathrm{enhancement}\;}{\mathrm{Signal}\;\mathrm{intensity}\;\mathrm{before}\;\mathrm{enhancement}}\times \;100\%$$

The slope of the time enhancement curve was determined using a simple linear regression formula. The following ratios were evaluated by the radiologist using TWIST MIP images and time intensity curves: the artery-lesion time (defined as the time interval between onset of enhancement in adjacent artery and onset of enhancement within vascular malformation) and the maximum lesion enhancement time (defined as the time interval between beginning of enhancement and maximum enhancement of the lesion).

### 2.4. Statistical Analysis

In statistical analysis, all tests were two-sided and *p* < 0.05 was considered statistically significant. All analyses were performed using STATISTICA (data analysis software system, v12, TIBCO software, Palo Alto, CA, USA).

The distribution of the continuous variables was examined with the Shapiro-Wilk test and they were found not to follow a normal distribution. They were therefore presented as medians and interquartile ranges (IQRs). The Mann-Whitney U-test was used to compare medians between two groups (high- and low-flow vascular malformations).

Categorical data were presented as counting (n) and percentages. The chi-squared test or exact Fisher’s test, as appropriate, were used to find significant differences between groups. u-Gauss test was used to compare percentages.

A receiver operating characteristic (ROC) curve was done to examine the performance of the artery-lesion time, the maximum lesion enhancement, the slope, and the maximum percentage increase of SI in the classification of the type of VM. The optimal cut-off was estimated according to the Youden index. Areas under curve (AUC) were reported with the corresponding confidence intervals (CIs). The values of AUC were compared using the DeLong test.

## 3. Results

Ninety patients were included in the study, 36 men (40%) and 54 women (60%). The median age was 30 years (IQR = 23–39).

### TWIST Quantitative Evaluation

**Figure 4:**
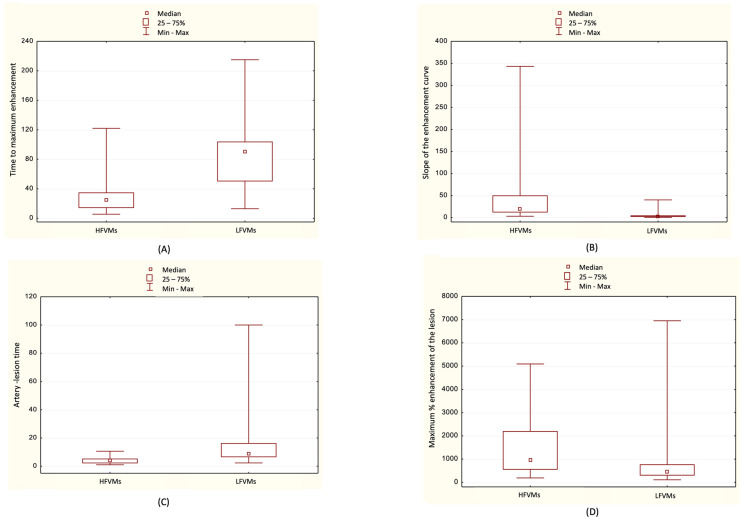
Box plots of calculated ratios according to the hemodynamic characteristics of vascular malformations (VMs). (**A**) Time to maximum enhancement. (**B**) Slope of the enhancement curve. (**C**) Artery-lesion time. (**D**) Maximum percentage enhancement of the lesion. HFVMs—high-flow vascular malformations, LFVM—low-flow vascular malformations. Lower and upper box boundaries 25th and 75th percentiles, respectively, square inside box median, lower and upper error lines minimum and maximum, respectively.

The medians and IQRs of the values of calculated ratios are presented in Table 1. The differences between medians are demonstrated on the box plots (Figure 4). They were examined with a Mann-Whitney U-test and found to be significant, with *p* < 0.001 for each ratio.

**Figure 5:**
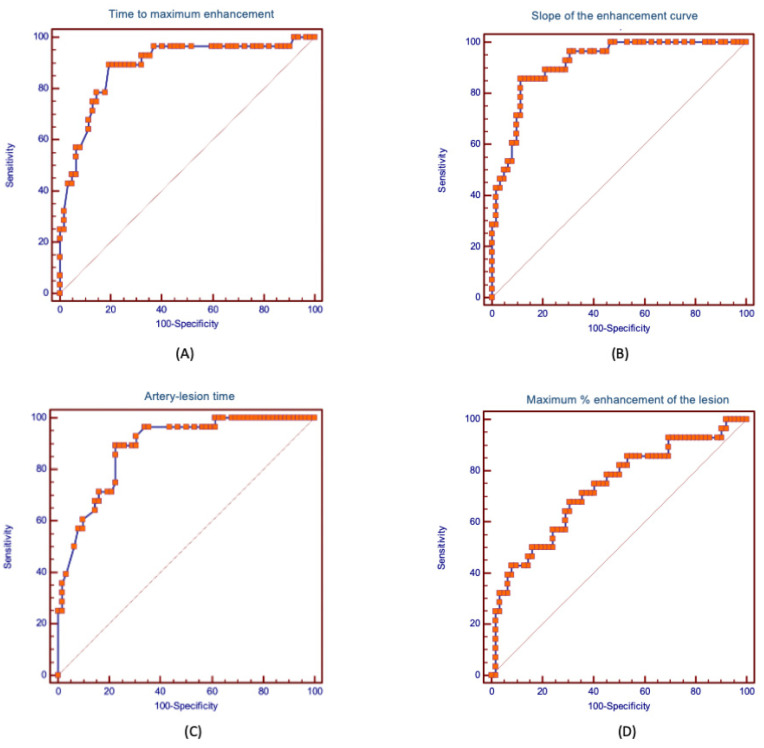
Receiver operating curves (ROCs) for: (**A**) time to maximum enhancement; (**B**) slope of the enhancement curve; (**C**) artery-lesion time; (**D**) maximum percentage enhancement of the lesion.

ROC analysis was performed for each variable to calculate the AUC and determine the optimal cutoff. The ROC curves are presented in Figure 5.

The results of ROC analyses for each variable, including values of AUC with the corresponding CIs, optimal cutoffs, specificity, sensitivity, as well as positive and negative predictive values are presented in Table 2. The AUCs for the following ratios were: for the slope of the enhancement curve AUC = 0.92 (CI 0.86-0.97), for the artery-lesion time AUC = 0.88 (CI 0.82–0.95), for the time to maximum enhancement AUC = 0.89 (CI 0.81–0.96), for the maximum percentage enhancement of the lesion AUC = 0.73 (CI 0.63–0.82). The optimal cut-offs for HFVMs were: for the slope of the enhancement curve > 8.7 s^−1^ (sensitivity of 86%, specificity of 89%), for the artery-lesion time ≤ 5.6 s (sensitivity of 89%, specificity of 77%), for the time to maximum enhancement ≤ 30 s (sensitivity of 94%, specificity of 100%), for the maximum percentage enhancement of the lesion > 662% (sensitivity of 68%, specificity of 69%).

## 4. Discussion

Dynamic contrast-enhanced magnetic resonance imaging (DCE-MRI) is a particularly important tool for evaluation of peripheral vascular malformations (PVMs). The distinguishing between high-flow vascular malformations (HFVMs) and low-flow vascular malformations (LFVMs) is crucial before choosing the proper treatment [2]. However, until now, only a few studies focused on establishing the quantitative criteria for this purpose, and they had considerable limitations. In this study, we propose the optimal cut-off values of different DCE-MRI ratios for such distinguishment.

The slope of the enhancement curve had the greatest diagnostic value (AUC = 0.92). The optimal cut-off value was of >8.7 s^−1^ for HFVMs, with the corresponding sensitivity of 86% and specificity of 89%.

These results suggest that this ratio can be very useful in daily practice. Interestingly, until now, only one previous study by Kociemba et al. [11] had investigated it, and the values of sensitivity and specificity were even higher (both of them of 100%). Nonetheless, their sample size was rather small (25 patients, including only 7 patients with HFVMs), which causes uncertainty about its representativeness for the general population.

The artery-lesion time and the time to maximum enhancement were previously examined in the studies of Ohgiya et al. [10] and Kociemba et al. [11]. Ohgiya et al. [10] found the cut-off values for HFVMs: of ≤5.0 s for artery-lesion time (sensitivity of 100%, specificity of 100%) and of ≤30 s for time to maximum enhancement (sensitivity of 100%, specificity of 60%). Similar cut-off values were determined by Kociemba et al. [11]: of ≤4.1 s for artery-lesion time (sensitivity of 100%, specificity of 57%) and of ≤27s for time to maximum enhancement (sensitivity of 94%, specificity of 100%).

In this study, the optimal cut-off value for artery-lesion time was close to those in the literature (≤5.6 s, sensitivity of 89%, specificity of 77%). The difference was in the time to maximum enhancement (in this study ≤40 s, sensitivity of 89%, specificity of 81%). The small size of samples in the aforementioned studies can be, again, the reason for this dissimilarity. The groups of Ohgiya et al. [10] and Kociemba et al. [11] contained 16 patients and 25 patients, respectively, versus 90 patients in this study. Moreover, Ohgiya et al. [10] analyzed DCE-MRI only in one slice without using maximum intensity projection (MIP) reconstructions.

Maximum percentage enhancement of the lesion had the lowest diagnostic value among examined ratios (AUC = 0.73). Its high value of optimal cut-off point (>662%) seems to be sensible, as HFVMs enhance during the early arterial phase. The blood concentration of contrast in this phase is high, which leads to the intensive enhancement. Conversely, LFVMs fill with contrast during the venous phase, when its blood concentration is already lower. Adequately to the low value of AUC, the sensitivity and specificity of the cut-off value was also low (of 68% and 69%, respectively).

Only one study discussed this ratio before, and this was the paper of Hammer et al. [13]. They did not use it to differentiate between HFVMs and LFVMs, but to distinguish between LVFMs with and without arteriovenous fistula, and they got relatively high AUC = 0.85. However, such division of LVFMs is not included in the most commonly applied classification of the International Society for the Study of Vascular Anomalies (ISSVA) [14] and does not impact treatment. Indeed, the low value of the AUC which was obtained in our study is an argument against the usefulness of this ratio in the differentiation between HFVMs and LFMVs.

Our secondary goal was to evaluate how the presence of flow voids in the VM can be useful in the diagnosis. It may be an interesting marker as there is no need for contrast injection to perform the assessment. In this study, the presence of flow voids had low sensitivity for the detection of HFVM (of 43%) but its specificity was very high (of 90%). These results are consistent with the study of Ohgiya et al. [10], who found its sensitivity was 50% and specificity was 100%. In a more recent study, by Höhn et al. [9], both of these values were very high, with a sensitivity of 100% and specificity of 97%. The main cause of this discrepancy may have been the use of the 3T MRI system in the paper of Höhn et al. [9], which gives higher spatial and temporal resolution than the 1.5 T system used in this study.

The main limitation of our study is its retrospective character, as the prospective study would be obviously of greater value. Moreover, we used the 1.5 T system, and further research with a higher field strength would be required. It would also be interesting to run and discuss the multivariable classification models; however, it was beyond the scope of this study. Although the measurements taken by the researcher were largely consistent with those conducted by assigned radiologists during patients’ hospital stays, they were taken only once, and the intra-observer reliability was not measured. This is obviously another limitation and should be avoided in subsequent studies.

It is worth mentioning that the blood distribution differs in various parts of the body (e.g., trunk vs. foot) and this can bias the swiftness of enhancement of differently located PVMs. The examined group was inhomogeneous with respect to the locations of lesions, which may possibly be a cause for the high variability of analyzed parameters, especially in HFVMs. Therefore, it would be recommended to carry out subsequent, preferably multi-center, studies in larger and more homogeneous groups. It is possible that in such conducted studies the accuracies of analyzed parameters would be even higher.

## 5. Conclusions

In this study we researched the quantitative ratios obtained using DCE-MRI and assessed the cut-off values for distinguishing between HFVMs and LFVMs. The slope of the enhancement curve was the most informative ratio with the cut-off value being >8.7 s^−1^ for the HFVM.

We also examined the usefulness of the presence of flow voids in such differentiation. In our 1.5 T MRI system it had very high specificity and low sensitivity, which was consistent with the previous paper.

To summarize, MR examination with a DCE-MRI sequence is a valuable imaging method in the diagnostics of vascular malformation and may be considered as a test of choice.

## Figures and Tables

**Table 1:** Medians of the time to maximum enhancement, slope of the enhancement curve, artery-lesion time, maximum percentage enhancement of the lesion for high-flow vascular malformations (HFVMs) and low-flow vascular malformations (LFVMs).

Parameter	HFVMs	LFVMs	*p* Value *
time to maximum enhancement (s)	25.0 (IQR = 14.0–35.0)	90.0 (IQR = 50.0–104.0)	<0.001
slope of the enhancement curve	19.2 (IQR = 11.6–50.3)	2.7 (IQR = 1.8–4.9)	<0.001
artery-lesion time (s)	4.1 (IQR = 2.1–5.4)	8.8 (IQR = 6.4–16.4)	<0.001
maximum percentage enhancement of the lesion (%)	954.0 (IQR = 543.0–2208.0)	473.0 (IQR = 290.0–775.0)	<0.001

* A significant difference between HFVMs and LFVMs.

**Table 2:** Values of optimal cut-off values, sensitivity, specificity and AUCs for high-slope vascular malformations (HFVMs) for time to maximum enhancement, slope of the enhancement curve, artery-lesion time, maximum percentage increase of signal intensity of the lesion. The differences between AUCs were statistically significant (*p* < 0.001).

Parameter	Cut-Off Value	Sensitivity	Specificity	AUC	Confidence Interval
time to maximum enhancement	≤40 s	89%	81%	0.89	0.81–0.96
slope of the enhancement curve	>8.7 s^−1^	86%	89%	0.92	0.86–0.97
artery-lesion time	≤5.6 s	89%	77%	0.88	0.82–0.95
maximum percentage increase of signal intensity	>662%	68%	69%	0.73	0.63–0.82

## Data Availability

The data presented in this study are available on request from the corresponding author. The data are not publicly available due to the privacy issues.
